# Specific associations between fungi and bacteria in broncho-alveolar aspirates from mechanically ventilated intensive care unit patients

**DOI:** 10.1080/21505594.2022.2146568

**Published:** 2022-11-16

**Authors:** Arezoo Shajiei, Lei Liu, Jolien Seinen, Willem Dieperink, Sven Hammerschmidt, Jan Maarten van Dijl, Hermie J.M. Harmsen

**Affiliations:** a Department of Medical Microbiology and Infection prevention, University of Groningen, University Medical Center Groningen, Groningen, The Netherlands; b Department of Critical Care, University of Groningen, University Medical Center Groningen, Groningen, The Netherlands; c Department of Molecular Genetics and Infection Biology, Interfaculty Institute for Genetics and Functional Genomics, Center for Functional Genomics of Microbes, University of Greifswald, Greifswald, Germany

**Keywords:** Respiratory, mycobiome, 18S rRNA, culture-independent, mechanical ventilation, sputum

## Abstract

The detection of fungi in the human respiratory tract may represent contamination, colonization or a respiratory infection. To develop effective management strategies, a more accurate and comprehensive understanding of the lung fungal microbiome is required. Therefore, the objective of the present study was to define the “mycobiome” of mechanically ventilated patients admitted to an intensive care unit (ICU) using broncho-alveolar aspirate (“sputum”) samples and correlate this with clinical parameters and the bacterial microbiota. To this end, the mycobiome of 33 sputum samples was analyzed by Internal Transcribed Spacer2 (ITS2) amplicon sequencing of the ribosomal operons. The results show that in the investigated sputa of mechanically ventilated patients *Candida* spp. were most frequently detected, independent of pneumonia or antimicrobial therapy. The presence of *Candida* excluded in most cases the presence of *Malassezia*, which was the second most-frequently encountered fungus. Moreover, a hierarchical clustering of the sequence data indicated a patient-specific mycobiome. Fungi detected by culturing (*Candida* and *Aspergillus*) were also detected through ITS2 sequencing, but other yeasts and fungi were only detectable by sequencing. While *Candida* showed no correlations with identified bacterial groups, the presence of *Malassezia* and *Rhodotorula* correlated with oral bacteria associated with periodontal disease. Likewise, *Cladosporium* correlated with other oral bacteria, whereas *Saccharomyces* correlated more specifically with dental plaque bacteria and *Alternaria* with the nasal-throat-resident bacteria *Neisseria*, *Haemophilus* and *Moraxella*. In conclusion, ITS2 sequencing of sputum samples uncovered patient-specific lung mycobiomes, which were only partially detectable by culturing, and which could be correlated to specific nasal-oral-pharyngeal niches.

## Introduction

Respiratory infections are a leading cause of morbidity and mortality worldwide, and, especially the acute exacerbations, represent a huge clinical and economic burden. It has been proposed that the microbiota of the respiratory tract, including both bacteria and fungi, could act as a gatekeeper that provides resistance to colonization by lung pathogens [1, 2]. The lung microbiota may even modulate viral infections and, moreover, viral and bacterial host interactions in the respiratory tract are believed to play key roles in lung immunity [1].

Our current understanding of the course of lung infections largely relies on culture-dependent analyses with a major focus on bacterial pathogens [3, 4]. On the other hand, the fungi in the respiratory tract, collectively referred to as the respiratory mycobiome, seem to represent an underappreciated component of the human microbiome [5]. This is remarkable in view of the fact that the involvement of fungi in pulmonary diseases can severely complicate patient management [6, 7]

The detection of fungi in the respiratory tract may relate to infection, colonization or contamination and the different scenarios require a very different patient management and have different prognosis [8]. The fungal microbiota is often altered in a state of disease and the functional consequences of such fungal dysbiosis on health and disease are still poorly understood [9]. Most studies on respiratory diseases indicated that a reduced fungal diversity correlates with a poorer lung function [10, 11]. This lower diversity could be attributed to an excessive growth of a single fungal species and/or the elimination of other micro-organisms [12]. Furthermore, prolonged therapy with antibiotics and the use of drugs, such as corticosteroids, may facilitate fungal growth [13]. Despite the critical roles of fungi, the research on the lung mycobiome is still in its infancy, which is at least in part due to technical limitations [14].

In healthy individuals, the microbial community of the lower respiratory tract mostly resembles that of the oral cavity and the upper respiratory tract, but the abundance of micro-organisms is significantly lower [1, 15]. This situation may change in patients, as exemplified in mechanically ventilated critically ill patients in an intensive care unit (ICU) where, due to medical interventions and the administration of drugs, more oral microbiota may enter the lower respiratory tract, thereby creating imbalances in the microbial equilibrium of the respiratory tract. This is due to several factors, such as a disabled cough reflex, impaired mucociliary clearance and the presence of an endotracheal tube. This dysbiotic state may ultimately lead to infections, such as pneumonia [3], [16–19].

In a previous study that was aimed at identifying the sources of antimicrobial activities in the lungs of mechanically ventilated patients in an ICU of the University Medical Center Groningen (UMCG), we investigated the bacterial content of 33 broncho-alveolar aspirates, here referred to as sputa [20]. It should be noted that, in this previous study, no control samples from healthy individuals could be included because of medical-ethical and technical considerations. The results of 16S rRNA gene sequencing showed that 27 of the investigated samples harboured no less than 635 bacterial species, and that the different sputum microbiomes were highly heterogeneous. *Streptococcus thermophilus, Staphylococcus epidermidis*, and *Streptococcus mitis* were the most frequently identified bacteria. The 16S rRNA gene sequencing furthermore showed that the bacterial compositions of different samples from the same patient were in most cases very similar, whereas the sputum microbiome of only two patients was found to change over time. No significant correlation could be detected between the sputum microbiome and antimicrobial activity [20]. Overall, this study was in line with previous studies confirming the non-sterile nature of the lung [21, 22]. Importantly, our previous study did not take into account possible contributions of the mycobiome to the overall composition of the sputum microbiota. For instance, one could expect that the fungal diversity might be lower upon antibiotic administration or that mechanical ventilation would lead to a dysbiotic microbiota with an increased fungal representation [3]. Therefore, the aim of the present study was to investigate the sputum mycobiome from mechanically ventilated patients and to assess possible associations with the bacterial microbiota or patient characteristics.

## Materials and methods

### Patient data and sputum collection

The current study is complementary to our previous investigation, where sputa were collected in the period between February and August 2015 from mechanically ventilated patients admitted to the department of Critical Care at UMCG [20]. All investigated sputa were collected as part of the normal care for mechanically ventilated patients as detailed by Seinen et al. [20]. All patients were subject to selective digestive tract decontamination (SDD) to prevent secondary colonization with Gram-negative bacteria, *S. aureus* and yeasts, which involved (i) application of non-absorbable antimicrobial agents (i.e. tobramycin, colistin, and amphotericin B) in the oropharynx and gastrointestinal tract, and (ii) pre-emptive systemic administration of cephalosporins (especially cefotaxime) [23]. Patients were excluded from the study upon suspected or diagnosed tuberculosis, suspected or diagnosed fungal or viral infections, immunodeficiency, administration of cytostatic agents, or positive end expiratory pressure >10 cm H_2_0. Diagnostic culturing was performed according to the standard diagnostic routine at UMCG within 24 h after sampling. Ethical approval for this study was obtained from the Medical Ethical Committee of the UMCG (research project number 2014.309), which decided that informed consent was not necessary because all patients admitted to the UMCG are informed that their data and (diagnostic) waste materials can be used for scientific research. All patient data and samples were collected with adherence to the Helsinki Guidelines and processed anonymously.

### DNA isolation and sequencing

Sputum aliquots of 100 µl were used for total DNA extraction with the Zymo Quick DNA kit (Zymo Research, CA, USA) as described previously [15]. A liquid culture of *Candida albicans* served as a positive control, and the kit ingredients were used as a negative control. Polymerase chain reaction (PCR) amplification, PCR cleanup, MiSeq library preparation and sequencing with an Illumina MiSeq System (Illumina Inc. San Diego, USA) were performed as described by Heida et al. 2016 [24]. Briefly, PCR on sputum-extracted DNA was performed with the forward primer FU3 and one of the 24 bar-coded primers per sample based on the reverse primer FU4 to amplify the fungal ITS2 region, as presented in Supplementary Table 1. The fungi-specific PCR resulted in amplified DNA products with a length of around 345 bases [25, 26]. These amplicons were sequenced with an Illumina MiSeq instrument using a 2 × 300 cartridge (Illumina, Eindhoven, the Netherlands). The resulting sequences were analyzed with publicly available software tools, including Trim Galore, QIIME 1.9.0 and BWA, alongside the R package Phyloseq [27], to produce a high-quality operational taxonomic unit (OTU) Table. Taxonomic information was assigned to each OTU using the Ribosomal Database projects (RDP) naive Bayesian classifier, which was trained with the UNITE database [28]. To minimize bias while using ecological measures, eight samples with less than 0.1% of total reads of all samples were removed from further analysis. Heatmaps were generated by using R package version 3.3.3 and edited with Adobe Illustrator CC 2017.

### Quantitative PCR

The total fungal load was estimated using a modified TaqMan-based qPCR assay with FungiQuant primers targeted at the 18S rRNA gene as described before by Lui et al. [29]. The total bacterial load was estimated using a modified TaqMan qPCR assay based on primers for the 16S rRNA genes described by Pragman et al. [13]. The primers for fungal and bacterial qPCR are listed in Supplementary Table 2. For quantification, a calibration curve was prepared by amplification of the *C. albicans* 18S rRNA gene that had been cloned into a TOPO TA cloning vector, purified, quantified and diluted down to a working concentration of 2 × 10^7^ copies per µl. As a control for quantification of 16S rRNA gene amplification, chromosomal DNA of *E. coli* DH5α was used with a genomic weight of 7 fg/cell and including seven ribosomal operons with 16S rRNA genes.

### Statistical analyses

Statistical analyses, including Spearman’s correlation tests and visualization of the results, were performed with the “Hmisc,” “psych,” “corrplot” and “ggplot2” packages of R (version 4.0.2). A p-value ≤0.05 was considered statistically significant. P-values were adjusted for multiple testing using the Benjamini-Hochberg method.

## Results

### Identification of sputum microbiomes from mechanically ventilated ICU patients

**Figure 1.**
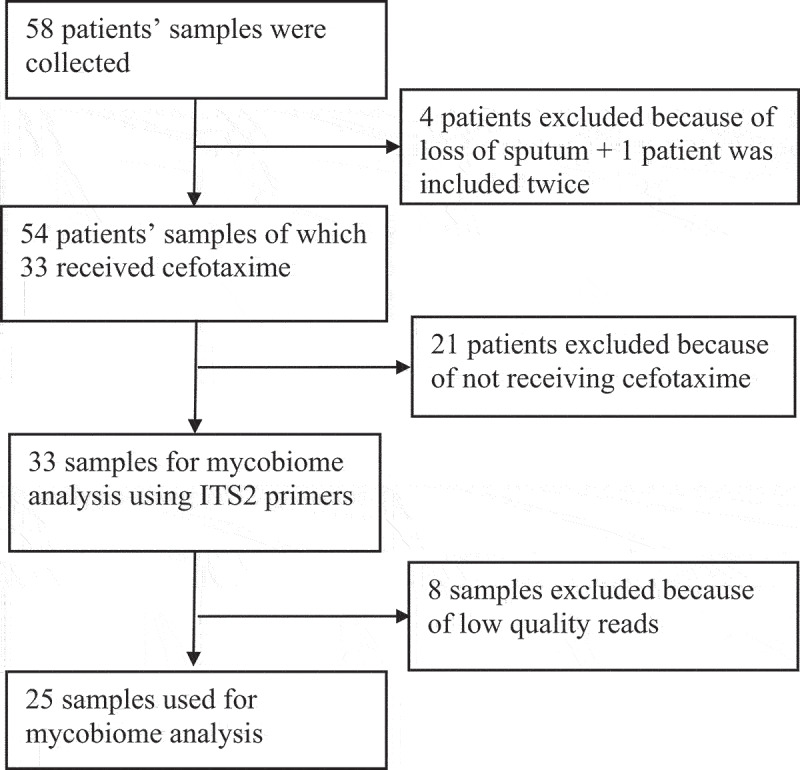
Flowchart of study design, patients and sample inclusion.

**Figure 2.**
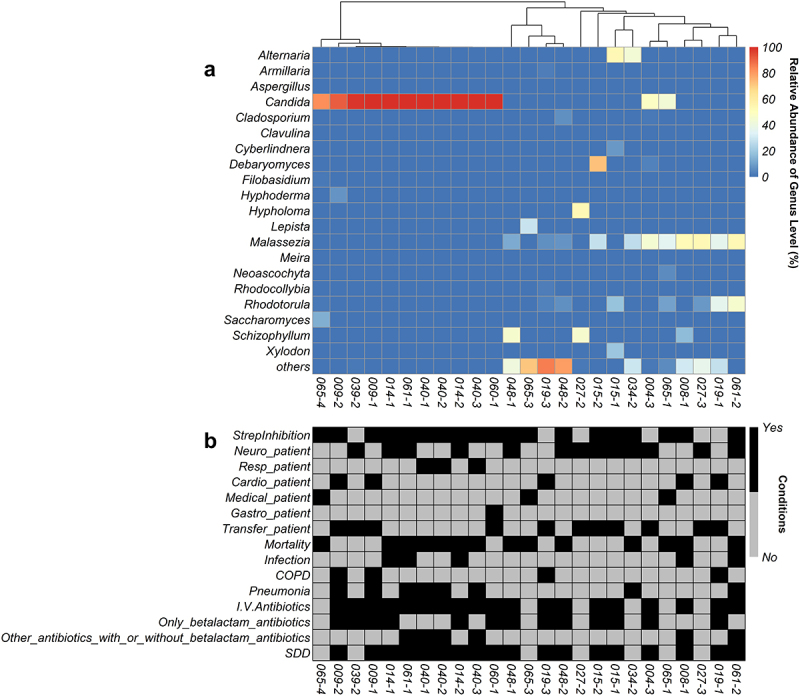
Heatmap of fungal abundance in sputum samples. Panel a shows the heatmap that was sorted based on a hierarchical clustering solution (Euclidean distance metric and average linkage) of the sputum mycobiome samples (n = 25). Rows represent the genera identified by ITS2 sequencing, and columns represent individual sputum samples. The colour key for relative abundance of the different genera is presented on the right of the heatmap. Panel B below the heatmap presents particular patient characteristics as recorded previously, where black indicates “yes” [20]. in particular, the following characteristics are included: StrepInhibition, a patient’s sputum was shown to inhibit streptococcal growth; Neuro, patient with neurological diagnosis; Resp, respiratory diagnosis; Cardio, cardiological diagnosis; Medical, medical diagnosis; Gastro, gastroenterological diagnosis; Transfer, ICU outcome was hospital transfer; Deceased, ICU outcome patient deceased; Infection, patient suffered infection; COPD, patient diagnosed with chronic obstructive pulmonary disease; Pneumonia, patient suffered from pneumonia; I.V. antibiotics, antibiotics were administered intravenously; Only_betalactam_antibiotics, patient received only beta-lactam antibiotics; Other antibiotics with or without betalactam antibiotics, patient received various other antibiotics; SDD, patient received selective decontamination of the digestive tract (SDD) according to the standard protocol of the UMCG [23]. a negative control, a water extraction control and a workbench swab extraction control were checked by PCR. However, these control PCRs did not result in amplicons and were therefore not sequenced. Additional bar plots of the fungal species abundance per sputum sample are shown in Supplementary figure 1.

For analyses of the fungal content of sputum samples, we used a set of previously collected samples from an initial cohort of 58 included mechanically ventilated ICU patients [20]. In this previous study, 33 sputum samples of 14 patients had been used to investigate the bacterial microbiomes by 16S rRNA gene sequencing, as indicated in the flow chart in Figure 1. Because the present study was aimed at investigating the fungal complement in these 33 samples, they were now used for PCR amplification and subsequent sequencing of 18S rRNA genes. PCR amplification of the 18S rRNA genes was successful in all 33 samples, but 8 samples yielded too low read numbers after sequencing that did not pass the threshold of 0.1% of the total reads of all samples. In the remaining 25 samples a total of 63 fungal species were identified belonging to 20 different genera. The heat-map based on these genera shows the relative abundance of all these samples Figure 2a. Overall, *Candida* and *Malassezia* were the most frequently identified resident fungal genera in the sputa, although both were rarely found in the same sample. From several patients multiple samples were collected at different time points, showing variations in the mycobiome composition (Supplementary figure S1A). Hierarchical clustering of the data showed that multiple samples from the same patient mostly clustered together (Figure 2a). In particular, Figure 2a shows that 11 samples contained mainly *Candida* species. An overview of these identified *Candida* species is depicted in Supplementary figure S1B, showing a dominance of *C. albicans* followed by *C. tropicalis*. Interestingly, from patients 065 and 061, respectively, 3 and 2 sputum samples were obtained on different time points. The mycobiome of these samples showed a completely different genus composition, some being dominated by *Candida* and some by other fungi as documented in Supplementary figure S1A. In addition, some fungal species that were detected by routine diagnostic culturing, especially *Candida* and *Aspergillus*, were also detected through ITS2 sequencing (Table 2), while other yeasts and fungi detected by ITS2 sequencing remained undetected by culturing (Supplementary Table 3). In addition, the alpha diversity based on the Shannon and other diversity indexes, and the beta diversity based on the Bray-Curtis distance were analyzed. This showed that the detection of *Candida* correlated with a low phylogenetic diversity (PD_whole_tree, R = −0.51 and P = 0.0085), while the detection of *Malassezia* correlated with high Shannon diversity (R = 0.52; P = 0.008) (Supplementary figure S2). Principal coordinate analysis (PCoA) of the beta diversity showed that the PCo1 was negatively correlated with the detection of *Candida albicans* (Supplementary figure S3).

## Relationships between mycobiome and patient data

As evidenced in Figure 2b, no significant correlation between the identification of fungal species and the recorded patient data, such as the disease status, antimicrobial therapy, lung diseases or ICU survival, was detectable. The abundance of the identified fungal species was also analyzed for possible correlation with patient data, but the number of correlations that were found was limited (Supplementary Table 4). However, it is noteworthy that negative correlations were observed between the detection of *Candida* species and the inflammatory markers C-reactive protein (CRP) and the sputum leukocyte count. On the other hand, a positive correlation was detected between the presence of *Alternaria* and CRP and the highest leukocyte counts of the patients measured during ICU admission.

## Inter-species fungal correlations and fungal-bacterial correlations

**Figure 3.**
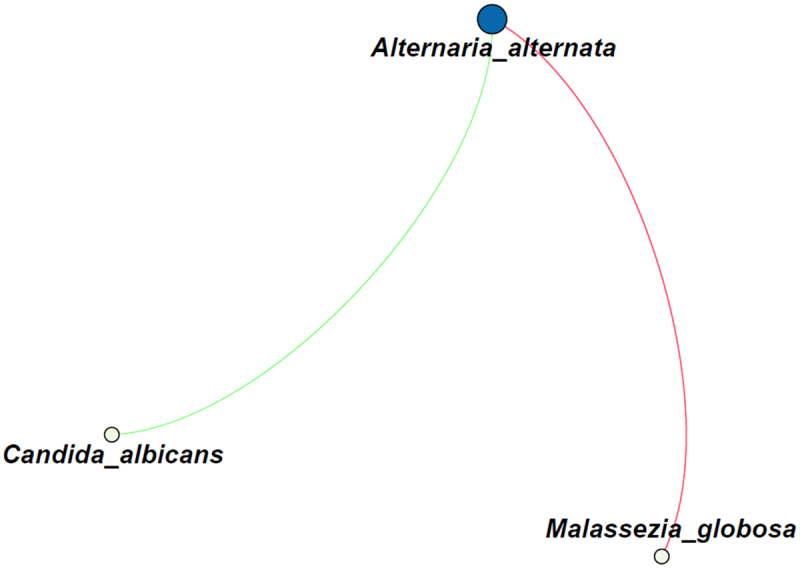
Correlation analysis of the fungal species identified in all 25 sputum samples. The green line indicates a negative correlation (R = −0.50), whereas the red line indicates a positive correlation (R = 0.54). The size of the circles indicate the number of connections. Only correlations with a p-value <0.05 are shown.

Possible correlations between fungi were analyzed on the species level and by performing a network analysis, which revealed a small network of correlations (Figure 3). The presence of *Alternaria alternata* was positively correlated with that of *Malassezia globosa* (R = 0.54), but negatively correlated with *C. albicans* (R = −0.50).

**Figure 4.**
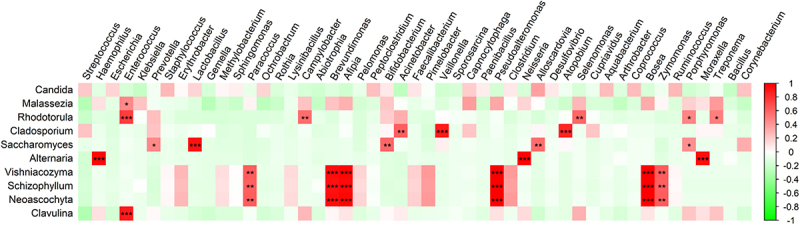
Spearman correlation heatmap between fungal and bacterial genera. Colors indicate the strength of the correlation as indicated by the colour bar at the right, and the stars show the significance of the correlation. *, p < 0.05; **, is p < 0.01; ***, p < 0.001.

Possible correlations between the detection of fungi and bacteria were also analyzed. Due to the low number of samples, only few correlations were detectable from this analysis (Figure 4), which makes it difficult to draw strong conclusions. Nonetheless, some correlations are noteworthy. For instance, the detection of *Candida* species trended to be weakly correlated with the detection of many different bacterial species, but none of these correlations was statistically significant. Interestingly, these weak correlations of *Candida* and bacterial species did not overlap with bacterial correlations found for *Malassezia* and *Rhodotorula* species. In particular, the detection of *Rhodotorula* correlated with oral bacteria that are associated with periodontal disease such as *Enterococcus* and *Porphyromonas* species [30], while the detection of *Cladosporium* correlated with other oral bacteria, such as *Veillonella*. The identification of *Saccharomyces* correlated with the detection of typical dental plaque bacteria, such as *Lactobacillus* and *Alloscardovia* species [31]. Lastly, the detection of *Alternaria* correlated with bacteria that seem to come from the nose- and throat area, especially *Neisseria*, *Haemophilus* and *Moraxella* species.

## Discussion

In this study, we analyzed the respiratory mycobiome based on sputum samples from mechanically ventilated ICU patients. The results showed that *Candida* is a dominant component of the mycobiome of various intubated and mechanically ventilated ICU patients, irrespective of antibiotic therapy or other patient characteristics. In fact, the detection of none of the identified fungal species could be correlated with the available clinical patient data. Furthermore, the presence of *Candida* species was not concurrent with species of the second most detected fungal genus *Malassezia*, as species belonging to these two genera seemed to exclude each other nearly completely.

*Candida* was the most frequently identified fungal genus in the sputum samples. *Candida* species are known as opportunistic pathogens, and they represent the most commonly isolated fungal genus in humans [9]. In contrast, it was shown previously that in samples of lung biopsy tissues with no dominantly abundant bacterial species, *M. globosa* is often dominant over other fungal species [32]. However, it should be noted that *M. globosa* is not considered as a human pathogen, because it is known to belong to the normal mycobiota of the human skin [32, 33]. The dominance of *Candida* within the respiratory mycobiome of ventilated ICU patients at the UMCG as judged from the analysis of their sputa is reminiscent of previous investigations, which reported *Candida* as being part of the fungal microbiota of various intubated and mechanically ventilated ICU patients [22,34–36]. *Candida* is also known to readily form biofilms on tubing and silicon voice prostheses [25], which could explain the observed prevalence in ventilated patients at least in part. Furthermore, admission of patients to the ICU and the administration of antimicrobial therapy is known to cause shifts in the fungal microbiota of the lower respiratory tract toward a prevalence of *Candida* [34]. Indeed, in other studies bacterial antibiotic therapy and mechanical ventilation have been described as risk factors for *Candida* colonization and, in fact, numerous factors in critically ill patients can contribute to the population shift toward colonization with this fungus [4, 14‒22]. In addition, the duration of mechanical ventilation was found to be associated with a decrease in lung bacterial diversity, giving more space for the colonization by fungi [3]. Such dysbiosis of microbial communities in the respiratory tract was most profoundly observed in patients who developed ventilator-associated pneumonia (VAP) [17]. Despite showing this kind of dysbiosis, the currently investigated patients, whose mycobiome shifted toward *Candida*, did not show invasive candidiasis or any related pathology.

As the duration of mechanical ventilation was previously found to be associated with a dysbiotic respiratory microbiome [37, 38], it is interesting to point out that a 2.5-fold increase was previously reported for the mortality of patients diagnosed with *Candida* [3]. However, in our present study no such correlation was detectable. In our cohort, patients who did not survive hospitalization in the ICU, were significantly older, underwent shorter lengths of stay in the hospital, and displayed higher SAPS II and APACHE IV disease severity scores compared to patients who did survive hospitalization in the ICU [20]. In another study, a possible relation was shown between the mortality by *Candida* infection and an impaired barrier function of the lung epithelium [22]. In contrast to this report, we observed a negative correlation between the presence of *Candida* and CRP, which is indicative of fungal colonization rather than an infection caused by *Candida*. Other studies on mechanically ventilated patients showed that *Candida* was most prevalent and abundant within these patients [39]. Moreover, the prolonged therapy with antibiotics in previous studies may have facilitated fungal growth in general and *Candida* in particular [39, 40].

Importantly, correlating the mycobiome to the bacterial microbiota identified by our sputum analyses resulted in several remarkable observations. Firstly, the presence of especially *Rhodotorula* species correlated with the detection of oral bacteria associated with periodontal disease, indicating that their detection might have a relation with oral health. A similar trend was observed for *Malassezia* species, albeit that it was not significant. Likewise, the detection of *Cladosporium* correlated with other oral bacteria, whereas the identification of *Saccharomyces* correlated with other specific dental bacteria. These observations imply that future research should include also the patients’ oral health status in order to clarify this correlation. Conversely, the detection of *Alternaria* species correlated with that of the nasal- and throat-resident bacteria *Neisseria*, *Haemophilus* and *Moraxella* [41]. Such correlations suggest an oral or nasal origin of the sputal fungi, which is seen more often in other studies [42, 43]. The observed similarities of the sputum microbiota with the oral or nasal microbiota can be explained by the patients’ intubation for mechanical ventilation, which may facilitate microbial migration toward the lower respiratory tract, and the fact that the sputa were collected via the intubation tube. Lastly, a cluster of three identified fungal genera, namely *Vishniacozyma*, *Schizophyllum* and *Neoaschyta*, is correlated with environmental (water) alpha- and gamma-proteobacteria, of which *Pseudomonas* seems to be the most relevant representative. This could suggest that these fungi and bacteria are contaminants derived from hospital water or a sink [44–46], or from the water needed to humidify the air that was used to ventilate the respective patients. Of note, we do not regard the identified *Pseudomonas* bacteria as methodological contaminants as the known contaminants are usually beta-proteobacteria [47]. On the other hand, they are also not typical lung bacteria, which are usually dominated by *Prevotella, Veillonella, Streptococcus*, *Pseudomonas* and *Fusobacterium* species, and more rarely *Haemaphilus* and *Neiserria* species [48].

In the current study, we observed a dissimilarity between the fungal species that were predominantly detected by routine diagnostic culturing (i.e. *Candida* and *Aspergillus*) and the results from our ITS2 sequencing, which identified other yeasts and fungi that remained undetected by culturing, such as *Alternaria* and *Malassezia*. Similarly, other studies found a discordance between culture- and sequence-based microbiome analyses of patient samples, indicating that for a high-resolution mycobiome analysis molecular methods are indispensable [49, 50]. Culture-independent microbial detection methods thus provide a different perspective on bacterial and fungal diversity. On this basis, Pragman et al. proposed a new ecological theory that lung tissue microbiota closely reflects the bronchial, oral and nasal microbiota with some evidence of ecological drift occurring in the lung tissue [51]. Our present observations are indeed consistent with this view as we frequently detected oral and nasal microbiota in the sputa of ventilated patients. Although previous studies correlated *Candida* to the ICU environment, the detected *Candida* is more likely derived from the patient’s microbiome rather than from patient’s environment, since the air used for the ventilation of patients is filtered and contact with the environment is restricted. However, it is conceivable that the detection of *Candida* is related to the applied ventilation tubes, on which biofilms may form, and which connect the oral cavity with the lungs. On the other hand, *Vishniacozyma*, *Schizophyllum* and *Neoascochyta* species are correlated to the presence of bacteria, such as *Afipia* and *Brevundimonas* species, which are pathogens that are not obviously related to the microbiota of the human respiratory tract, and that may thus be of environmental or ICU origin [25, 52, 53]. A definite relationship, however, could not be established since all investigated sputa were from mechanically ventilated patients. Furthermore, all our sputum samples were collected by aspiration through the intubation tube. Consequently, contamination of samples from oral or pharyngeal microbiota may have occurred. Extending these studies with bronchial lavage and brush samples may give more precise information on the lung microbiota, although oral and pharyngeal contaminations will remain hard to exclude [54, 55]. Another limitation of our present pilot study concerns the restricted number of samples and the absence of samples from healthy volunteers. However, it will be difficult, if not impossible, to aspirate sputum from non-intubated healthy volunteers. Nonetheless, the ITS2 sequence data did show a hierarchical clustering of multiple samples from the same patient, indicating patient-specific mycobiome patterns are not random. Despite this, only limited correlations were found with the patient characteristics, and an expansion of the patient cohort may reveal more sample clusters that connect with patient characteristics.

In conclusion, based on our present data, which were obtained from conventional microbiological culturing, ITS2 sequencing and qPCR, we conclude that *Candida* is the most encountered fungus in the investigated sputa, suggesting colonization of the lungs of the respective mechanically ventilated ICU patients. While the presence of Candida did not specifically correlate with other microorganisms, other identified fungal species showed relationships with oral and nasal microbiota, and microorganisms from environmental origin. The combined observations presented here show that the ecological characteristics that shape fungal communities within mechanically ventilated patients are complex. Lastly, it is evident from our data that culture methods alone do not lead to a full appreciation of the complexity of the lung mycobiome, and that culture-independent methods will most likely allow further improvements in the clinical management of fungi-associated respiratory diseases.

## Supplementary Material

Supplemental Material

## Data Availability

The sequencing data that support the findings of this study are openly available at NCBI at https://www.ncbi.nlm.nih.gov/bioproject/; BioProject ID: PRJNA878464 and SAMN30726734.
