# Supplementary material for: Specific associations between fungi and bacteria in broncho-alveolar aspirates from mechanically ventilated intensive care unit patients
Source: Virulence. 2022 Nov 16;13(1):2022–31. doi: 10.1080/21505594.2022.2146568 (PMC9673952; doi:10.1080/21505594.2022.2146568)
Supplement: Supplemental Material [file KVIR_A_2146568_SM6261.docx]

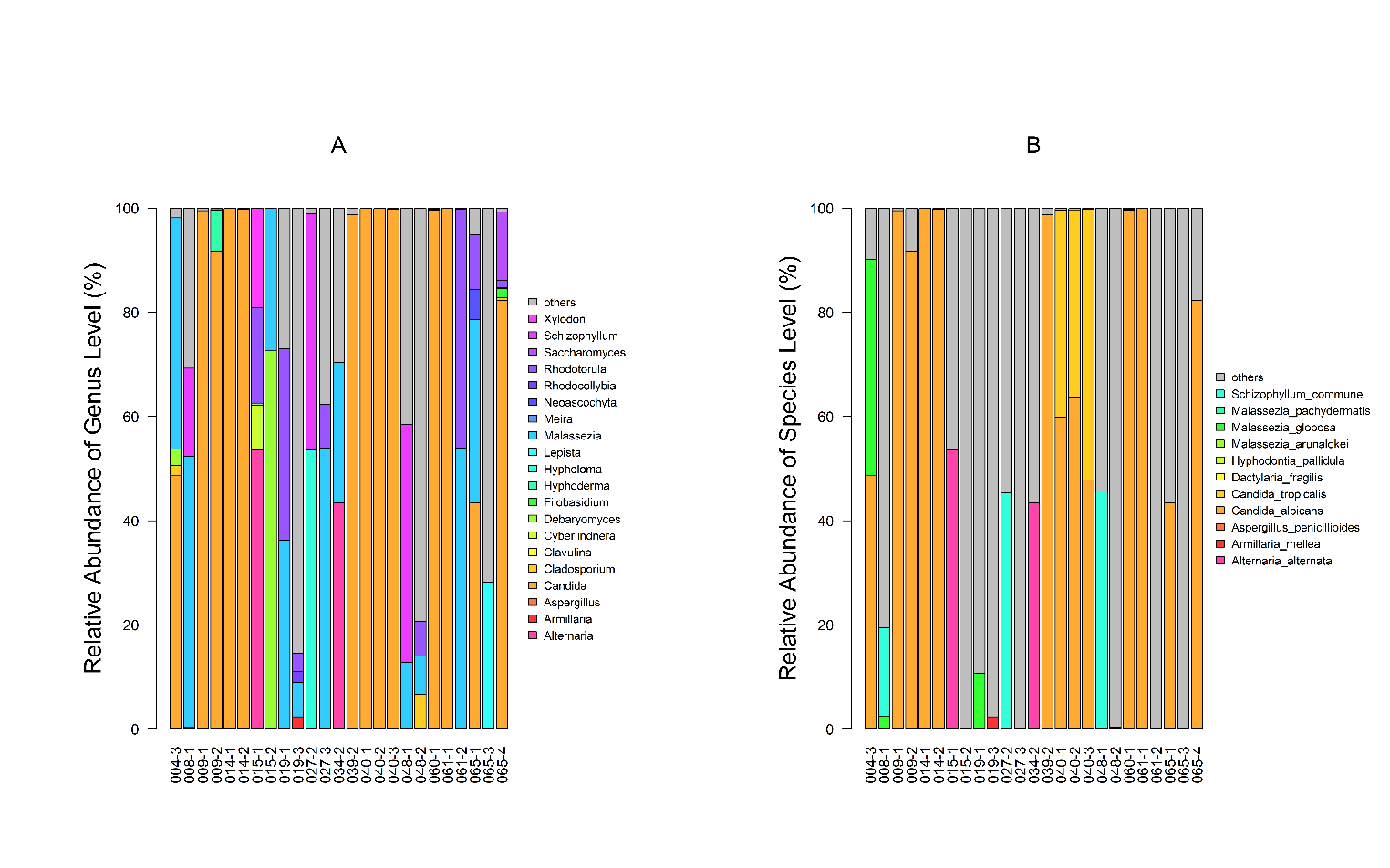

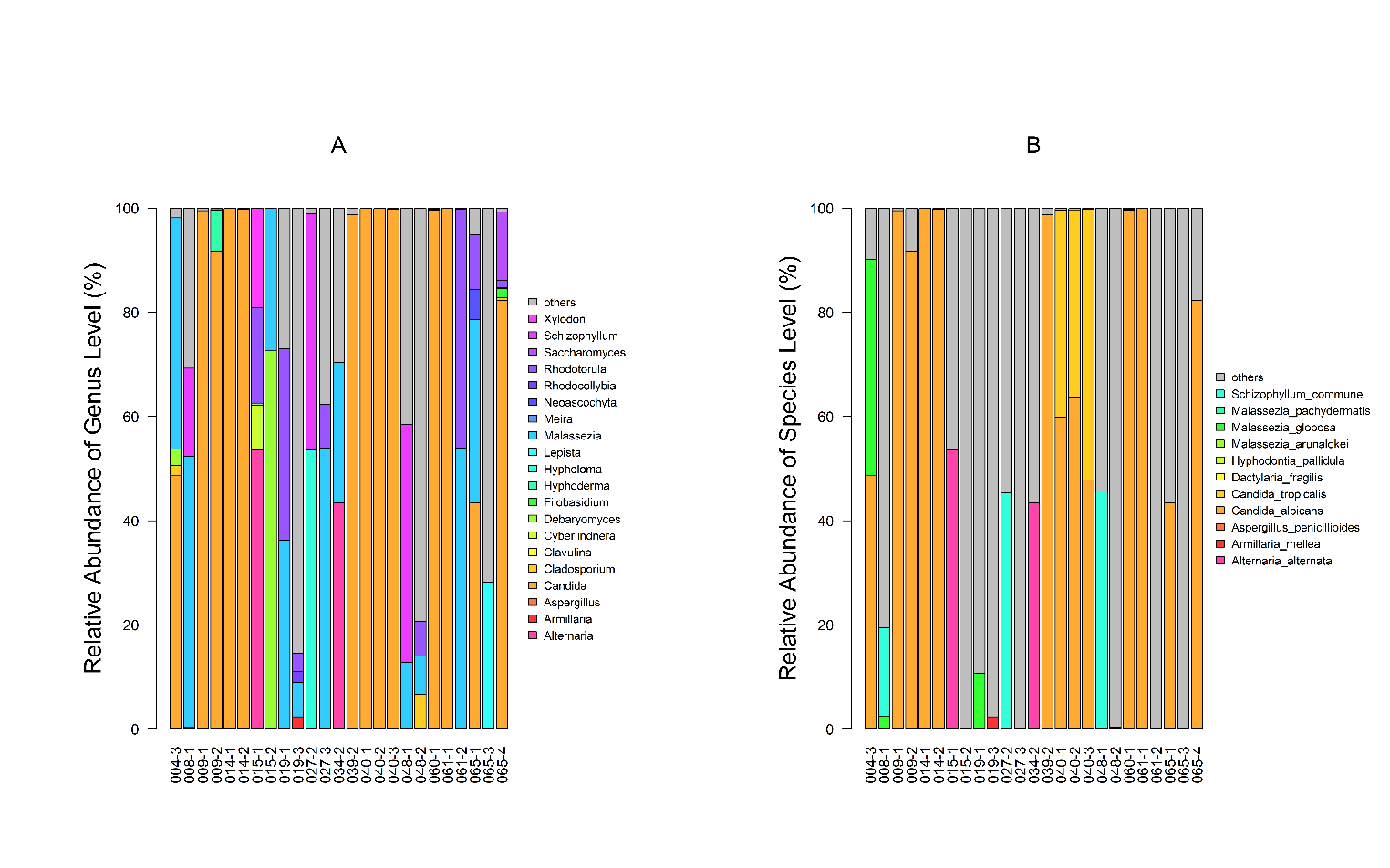


**Supplementary Figure 1. Bar plot of fungal abundance in sputum samples.** The sputum samples are indicated on the X-axis, and the relative abundance of particular identified fungal genera (**A**) or species (**B**) are shown individually on the Y-axis marked by color code. **A,** the top-20 most abundant fungal genera. **B,** the top-20 most abundant species. Due to the limited diversity of the ITS2 database, not all reads identified at the genus level could be related to specific species and such reads are therefore indicated as ‘others’.


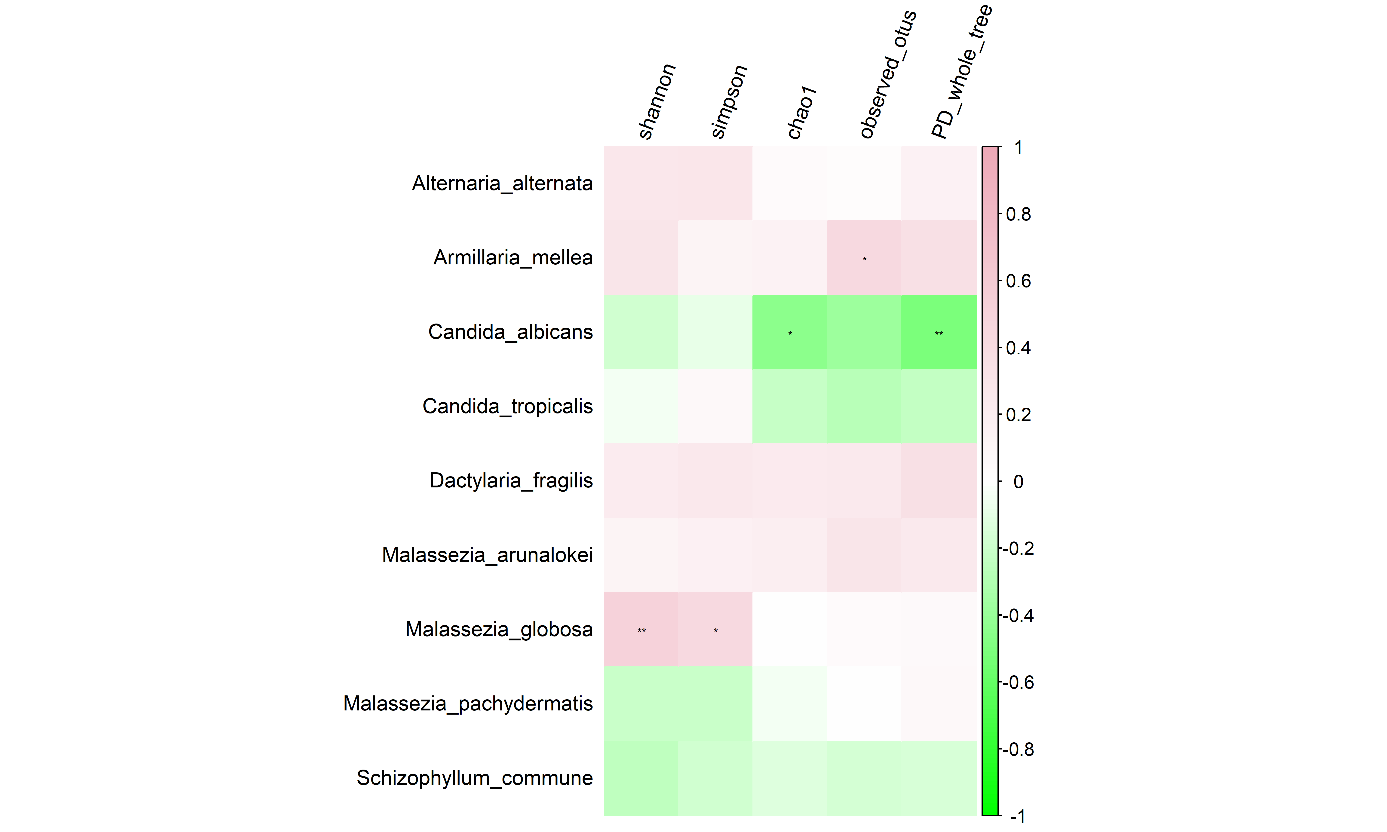


**Supplementary Figure 2.** Correlation heat-map between diversity indexes and the first nine most abundant fungal species. The stars indicate the significance of the correlation. *, p< 0.05; **, p< 0.01.


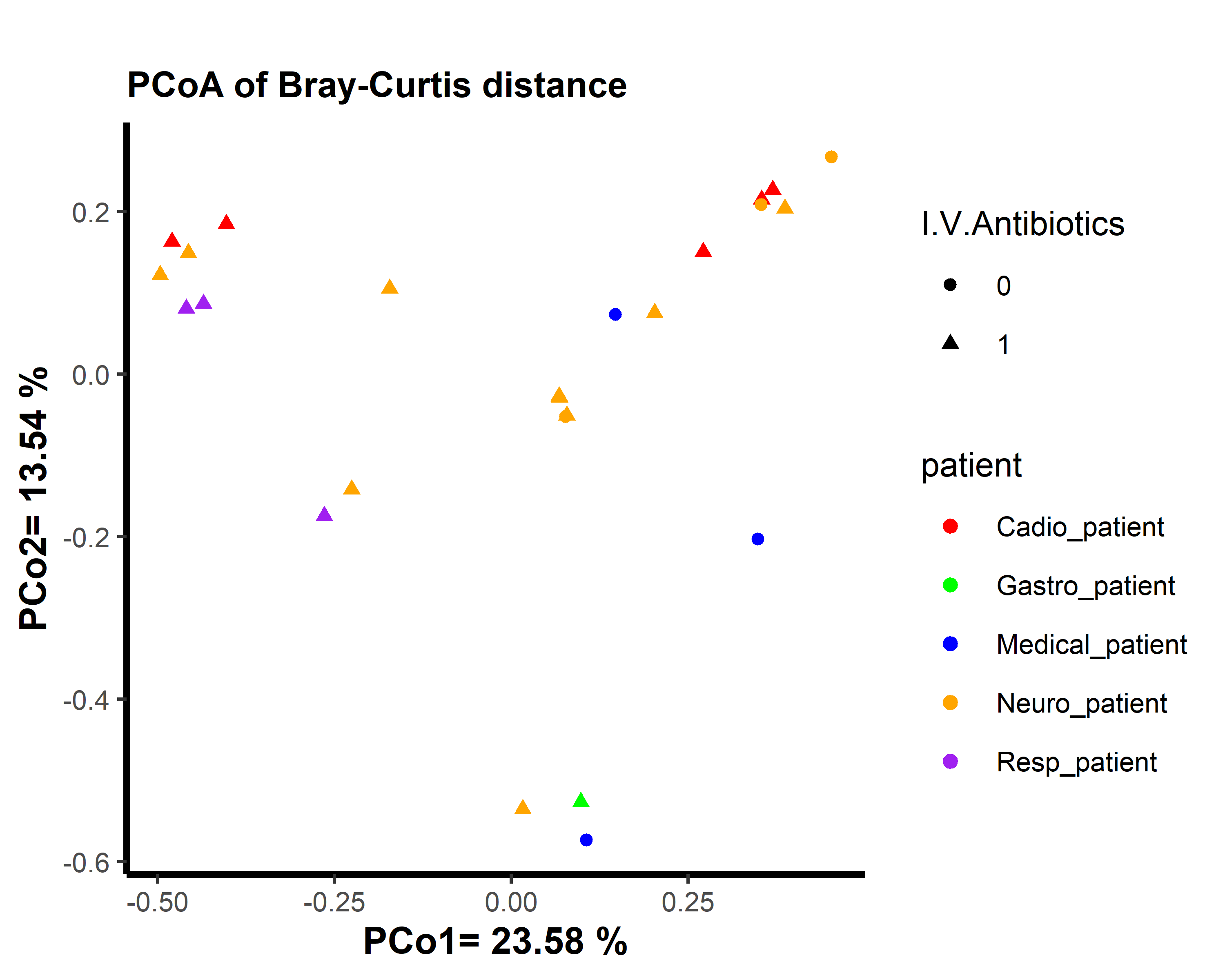


**A**


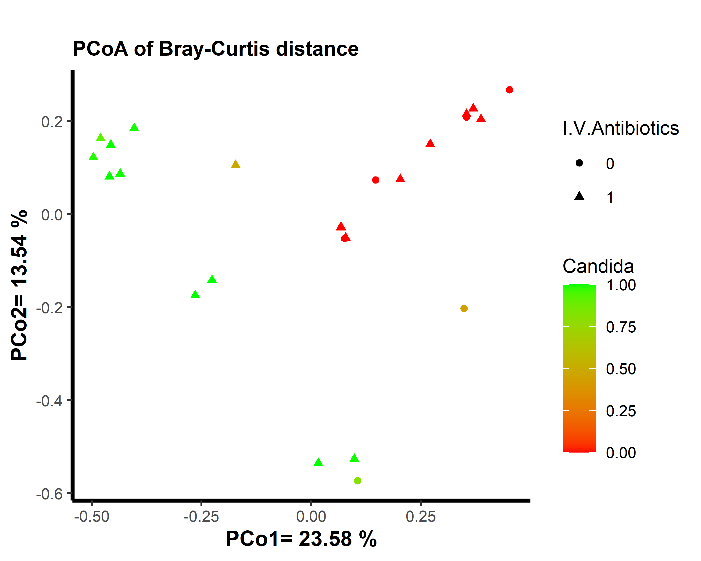

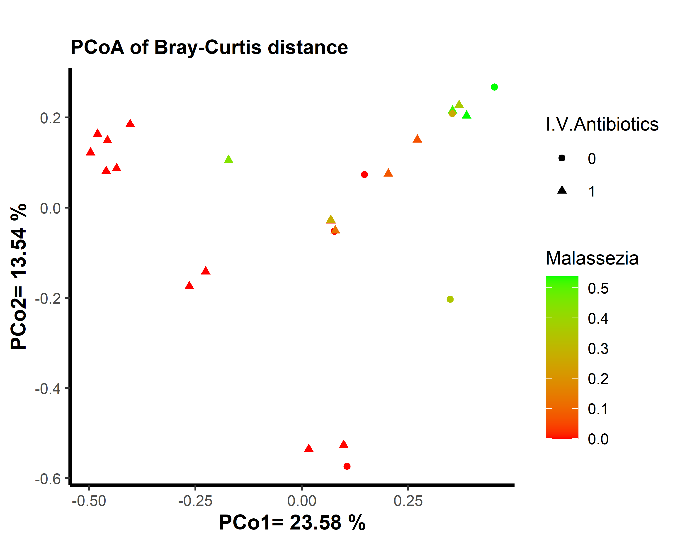


**C**

**B**

**Supplementary Figure 3.** PCoA analysis based on the Bray-Curtis distance of the mycobiome composition of sputum samples from ICU patients. The shape of the symbols indicates the intravenous (I.V.) administration of antibiotics. **A.** The sample colors refer to the patient’s diagnosis upon admission to the ICU. **B.** The sample colors refer to the relative abundant of *Candida*. **C.** The sample colors refer to the relative abundance of *Malassezia*.

**Supplementary Table 1.** Primers specific for fungi.

Nucleotide sequences of primers used in the construction of libraries for Illumina sequencing. Lowercase letters denote adapter sequences necessary for binding to the flow cell, underlined lowercase letters denote binding sites for the Illumina sequencing primers, and bold uppercase letters highlight the barcode index sequences.

| Fu3_F | aatgatacggcgaccaccgagatctacactctttccctacacgacgctcttccgatctNNNNGCATCGATGAAGAACGCAGC |
| --- | --- |
| Fu4_1R | caagcagaagacggcatacgagat**ATCTGC**gtgactggagttcagacgtgtgctcttccgatctTCCTCCGCTTATTGATATGC |
| Fu4_2R | caagcagaagacggcatacgagat**ACACGA**gtgactggagttcagacgtgtgctcttccgatctTCCTCCGCTTATTGATATGC |
| Fu4_3R | caagcagaagacggcatacgagat**AGGTTC**gtgactggagttcagacgtgtgctcttccgatctTCCTCCGCTTATTGATATGC |
| Fu4_4R | caagcagaagacggcatacgagat**CATGAC**gtgactggagttcagacgtgtgctcttccgatctTCCTCCGCTTATTGATATGC |
| Fu4_5R | caagcagaagacggcatacgagat**GCTATC**gtgactggagttcagacgtgtgctcttccgatctTCCTCCGCTTATTGATATGC |
| Fu4_6R | caagcagaagacggcatacgagat**GGACTT**gtgactggagttcagacgtgtgctcttccgatctTCCTCCGCTTATTGATATGC |
| Fu4_7R | caagcagaagacggcatacgagat**GGCAAT**gtgactggagttcagacgtgtgctcttccgatctTCCTCCGCTTATTGATATGC |
| Fu4_8R | caagcagaagacggcatacgagat**TCTCGG**gtgactggagttcagacgtgtgctcttccgatctTCCTCCGCTTATTGATATGC |
| Fu4_9R | caagcagaagacggcatacgagat**TCAGCG**gtgactggagttcagacgtgtgctcttccgatctTCCTCCGCTTATTGATATGC |
| Fu4_10R | caagcagaagacggcatacgagat**TGTGCC**gtgactggagttcagacgtgtgctcttccgatctTCCTCCGCTTATTGATATGC |
| Fu4_11R | caagcagaagacggcatacgagat**TGCACG**gtgactggagttcagacgtgtgctcttccgatctTCCTCCGCTTATTGATATGC |
| Fu4_12R | caagcagaagacggcatacgagat**AAGGCC**gtgactggagttcagacgtgtgctcttccgatctTCCTCCGCTTATTGATATGC |
| Fu4_13R | caagcagaagacggcatacgagat**ACCAGG**gtgactggagttcagacgtgtgctcttccgatctTCCTCCGCTTATTGATATGC |
| Fu4_14R | caagcagaagacggcatacgagat**AGCCTG**gtgactggagttcagacgtgtgctcttccgatctTCCTCCGCTTATTGATATGC |
| Fu4_15R | caagcagaagacggcatacgagat**AGCGAC**gtgactggagttcagacgtgtgctcttccgatctTCCTCCGCTTATTGATATGC |
| Fu4_16R | caagcagaagacggcatacgagat**CTACGC**gtgactggagttcagacgtgtgctcttccgatctTCCTCCGCTTATTGATATGC |
| Fu4_17R | caagcagaagacggcatacgagat**CTCCAG**gtgactggagttcagacgtgtgctcttccgatctTCCTCCGCTTATTGATATGC |
| Fu4_18R | caagcagaagacggcatacgagat**CCGTAG**gtgactggagttcagacgtgtgctcttccgatctTCCTCCGCTTATTGATATGC |
| Fu4_19R | caagcagaagacggcatacgagat**CGGTGT**gtgactggagttcagacgtgtgctcttccgatctTCCTCCGCTTATTGATATGC |
| Fu4_20R | caagcagaagacggcatacgagat**CGGAAC**gtgactggagttcagacgtgtgctcttccgatctTCCTCCGCTTATTGATATGC |
| Fu4_21R | caagcagaagacggcatacgagat**GTGCTG**gtgactggagttcagacgtgtgctcttccgatctTCCTCCGCTTATTGATATGC |
| Fu4_22R | caagcagaagacggcatacgagat**GAACGG**gtgactggagttcagacgtgtgctcttccgatctTCCTCCGCTTATTGATATGC |
| Fu4_23R | caagcagaagacggcatacgagat**GGATGC**gtgactggagttcagacgtgtgctcttccgatctTCCTCCGCTTATTGATATGC |
| Fu4_24R | caagcagaagacggcatacgagat**GGCGTA**gtgactggagttcagacgtgtgctcttccgatctTCCTCCGCTTATTGATATGC |

**Supplementary Table 2.** Nucleotide sequences of primers used in the qPCR for 18S and 16S rRNA gene amplification.

| **18S** |  |
| --- | --- |
| **FungiQuant-F** | 5′-GGRAAACTCACCAGGTCCAG-3′ |
| **FungiQuant-R** | 5′-GSWCTATCCCCAKCACGA-3′ |
| **FungiQuant-Prb** | (6FAM) 5′-TGGTGCATGGCCGTT-3′ (MGBNFQ) |
| **16S** |  |
| **Forward** | 27F 5'-AGA-GTT-TGA-TCM-TGG-CTC-AG-3' |
| **Reverse** | 1491R 5'-CGGYTACCTTGTTACGACTTC-3' |
| **Probe** | P535 5’-FAM- CAg CCg Cgg TAA TA -MGBNFQ -3’ |

**Supplementary Table 3.** Comparison of results from routine diagnostic culturing of patient sputa and the top-3 most abundant bacterial species identified by 16S and 18S rRNA sequence analysis of sputa from the same patient as investigated in the present study.

| Patient | Diagnostic culturing | Sample | 16S:  Total number of reads | 18S:  Total number of reads | 16S:  Percentage | 18S:  Percentage |
| --- | --- | --- | --- | --- | --- | --- |
| 004 | - | 004-3 | 23054 | 12583 | *S. anginosus* (41)  *C.* *gingivalis* (14)  *L. gasseri* (11) | *Candida 96*  *Debaryomyces 1*  *Filobasidium 1* |
| 008 | No growth | 008-1 (*) | 1514 | *12933* | *B. vesicularis* (13)  *Afipia* genosp. (11)  *E. aquimaris* (10) | *Irpex 50*  *Schizophyllum 27*  *Malassezia 22* |
| 009 | Aerobic Gram-positive mixed flora | 009-1 | 24239 | *157* | *S. pneumoniae* (57)  *S. mitis* (18)  *G. sanguinis* (8) | *Candida 100* |
|  |  | 009-2 | 24103 | *225* | *L. gasseri* (26)  *L. fermentum* (21)  *P. melaninogenica* (17) | *Candida 99*  *unidentified 1* |
| 014 | *E. coli*  Yeast  *A. fumigatus* | 014-1 | 24591 | *36* | *E. coli* (54)  *L. gasseri* (13)  *P. melaninogenica* (12) | *Acremonium 28*  *Lepista 25*  *Rhodotorula 14* |
|  |  | 014-2 | 22448 | *30* | *E. coli* (85)  *S. mitis* (6)  L. gasseri (2) | *Candida 100* |
| 015 | - | 015-1 (*) | 2593 | *98* | *S. sanguinis* (34)  *S. mitis* (13)  *P. melaninogenica* (12) | *Alternaria 68*  *Rhodotorula 27*  *Cyberlindnera 4* |
|  |  | 015-2 | 23781 | *194* | *R mucilaginosa* (37)  *S. sanguinis* (33)  *G. sanguinis* (11) | *Candida 94*  *Debaryomyces 4*  *Malassezia 2* |
| 019 | No growth | 019-1 (*) | 7538 | *9931* | *S. constellatus* (14)  *P. melaninogenica* (13)  *C. concisus* (10) | *Rhodotorula 48*  *Irpex 33*  *Malassezia 20* |
|  |  | 019-3 (*) | 3634 | *6701* | *L. xylanilyticus* (24)  *S. thermotolerans* (15)  *M. populi* (7) | *Cyathicula 66*  *Irpex 21*  *Rhodotorula 7* |
| 027 | *E. coli* | 027-2 | 18304 | *17* | *K. pneumoniae* (60)  *E. coli* (35)  *K. oxytoca* (4) | *Schizophyllum 65*  *Hypholoma 29*  *unidentified 6* |
|  |  | 027-3 | 21692 | *12183* | *K. pneumoniae* (62)  *E. coli* (33)  *K. oxytoca* (4) | *Irpex 31*  *Oberwinklerozyma 28*  *Malassezia 26* |
| 034 | Aerobic Gram-positive mixed flora | 034-2 | 22635 | *11457* | *H. influenzae* (90)  *N. meningitidis* (4)  *S. pneumoniae* (3) | *Alternaria 54*  *Malassezia 27*  *Oberwinklerozyma 19* |
| 039 | Throat flora |  |  |  |  |  |
|  |  | 039-2 | 21353 | *1066* | *G. sanguinis* (28)  *S. thermophilus* (21)  *H. parainfluenzae* (20) | *Candida 100* |
| 040 | Yeast | 040-1 (*) | 1015 | *2577* | *O. pseudintermedium* (17)  *E. aquimaris* (8)  *E. coli* (8) | *Candida 100* |
|  |  | 040-2 (*) | 451 | 715 | No reliable identifications | Candida 99  Xylodon 1 |
|  |  | 040-3 (*) | 2454 | *384* | *M. populi* (17)  *D. piger* (10)  *P. graminis* (8) | *Candida 100* |
| 048 | Throat flora | 048-1 | 21289 | *52* | *S. mitis* (65)  *G. sanguinis* (6)  *S. thermophilus* (5) | *Candida 46*  *Schizophyllum 46*  *Malassezia 7* |
|  |  | 048-2 (*) | 14581 | *97484* | *S. mitis* (42)  *P. melaninogenica* (11)  *A. para-adiacens* (6) | *Candida 86*  *Rhodotorula 9*  *Cladosporium 4* |
| 060 | No growth | 060-1 | 24537 | *6176* | *S. pneumoniae* (92)  *H. parahaemolyticus* (7)  *S. mitis* (1) | *Candida 100* |
| 061 | No growth | 061-1 (*) | 7545 | *707* | *S. mitis* (29)  *S. thermophilus* (17)  *S. epidermidis* (15) | *Candida 100* |
|  |  | 061-2 (*) | 11813 | *128* | *E. hirae* (71)  *E. faecalis* (23)  *E. durans* (2) | *Rhodotorula 58*  *Malassezia 42* |
| 065 | Yeast | 065-1 (*) | 237 | 27441 | No reliable identifications | Candida 52  Malassezia 27  Rhodotorula 11 |
|  |  | 065-3 (*) | 470 | 21087 | No reliable identifications | Oberwinklerozyma 75  Lepista 25 |
|  |  | 065-4 (*) | 386 | 45167 | No reliable identifications | Candida 87  Saccharomyces 10  Filobasidium 1 |
| Negative control –  Buffers used for DNA isolation and PCR |  | (*) | 326 |  | *E. coli* (20)  *E. aquimaris* (12)  *Afipia* genosp. (8) |  |
| Positive control –  *S. pneumoniae* TIGR4 culture |  |  | 20268 |  | *S. pneumoniae* (96)  *E. coli* (1)  *P. bivia* (1) |  |

Note that for most patients, diagnostic culturing was performed only once. In case culturing was performed more than once, the results of culturing on the date closest to the collection date of sputum sampling for the present study is presented. ‘-‘ means that no diagnostic culturing of the respective sputum was done; ‘no growth’ indicates that no microbial growth was detectable upon diagnostic culturing. Study sputum samples marked with an (*) yielded upon 16S rRNA sequencing >30% of reads relating to commonly encountered contaminants.

**Supplementary Table 4. Correlation matrix of clinical parameters with sputum mycobiota of mechanically ventilated of ICU patients.** Spearman’s tests were performed to identify significant correlations. Only significant correlations are presented (p ≤ 0.05).

|  | ICU lenght of stay | APACHE IV score | Leukocytes during 1st sputum collection | CRP during 1st sputum collection | | Lowest leukocytes during ICU admission | Highest CRP during ICU admission |
| --- | --- | --- | --- | --- | --- | --- | --- |
| *Aspergillus* | 0,47 | -0,59 | -0,34 | -0,48 | -0,48 | | - |
| *Candida* | - | - | - | - | - | | - |
| *Rhodotorula* | - | - | - | 0,35 | - | | - |
| *Cladosporium* | - | - | - | - | -0,34 | | - |
| *Alternaria* | - | - | - | - | 0,42 | | 0,35 |
| *Lepista* | - | - | - | - | - | | 0,34 |
